# Welcome to Volume 6 of *Concussion*

**DOI:** 10.2217/cnc-2020-0025

**Published:** 2021-04-30

**Authors:** Lucy Chard, Lauren Pulling

**Affiliations:** 1 Future Science Group Unitec House, 2 Albert Place, London N3 1QB, UK; 2 The Drake Foundation Unitec House, 2 Albert Place, London N3 1QB, UK

**Keywords:** biomarkers, chronic traumatic encephalopathy, concussion, epidemiology, injury, post-traumatic stress, team sport, traumatic brain injury

To all of our readers, Happy New Year and welcome to the first issue of Volume 6 of *Concussion*. Year 2020 was a tumultuous time for everyone and it involved many canceled plans and changes to how we work. However, we are proud to have been able to maintain our commitment to publishing the key advances in clinical and translational research across this niche, yet fast paced, area of research.

In this Foreword, we take a look back at some of our content highlights from 2020 as well as provide an update regarding the work of our parent organization, The Drake Foundation (London, UK) [1], on behalf of which the journal is published; looking at the projects they are currently funding in order to help further concussion research.

## Content highlights from 2020

As usual, the quality of articles published this year has been high and we thank all of our authors and reviewers for their hard work and excellent research.

Mirroring our Foreword from this time last year, once again our most-read article comes from a group at RightEye LLC (MD, USA) who, in a follow-up to their 2019 article, evaluated the ability of the eye-tracking tests to differentiate between different levels of traumatic brain injury (TBI) severity and healthy controls [2]. In their study, vertical smooth pursuit eye movements were used as a biomarker for TBI, with the group concluding that their eye-tracking technology was able to provide a timely and objective method of differentiating between TBI severity. Gaining an Altmetric score of 110, the article is in the top 5% of all research articles scored by the metrics system and is in the 97th percentile of all articles of the same age.

Our next most-read article is a Commentary that weighs up the pros and cons of team sport participation for today’s youth [3]. Lead author Scott L Zuckerman (Vanderbilt Sports Concussion Center, TN, USA) and colleagues discuss the benefits of team sport versus the increasing concerns of concussion and chronic traumatic encephalopathy felt by many parents. They conclude that participation in team sports is vital for the physical and mental health of younger generations, a conclusion endorsed by 17 expert concussion professionals. Expanding on the points made in the Commentary, Zuckerman partook in a Peek Behind the Paper interview on our partner website, Neuro Central [4]. If you enjoyed the published article, we recommend you read what he had to say.

Following the theme of team sports, our next article of note is a comprehensive review of the literature regarding concussion in soccer. In their paper, Mooney *et al*. (University of Alabama, AL, USA) review a total of 64 articles examining the epidemiology, injury mechanisms, sex differences, as well as the neurochemical, neurostructural and neurocognitive changes associated with soccer-related concussion [5].

Completing the list of our top five articles from 2020, we have two articles on concussion from the world of ice hockey. In the first, Asa Engstrom (Lulea University of Technology, Sweden) *et al*. investigate the long-term effect of concussion on the lives of ex-professional ice hockey players [6]. Including extracts from the interviews of nine former players, the article describes what suffering multiple concussions meant for players who had been forced to end their career due to their injuries. The research group found that many players felt that they had lost their identity as a player, and often struggled to develop an off-the-ice persona or find other sources of meaning in their lives. Engstrom was also an author on our final paper, this time with lead author Anna Gard (Lund University, Sweden) and colleagues [7]. Again looking at retired hockey players, in this paper the group used the Sports Concussion Assessment Tool 5th Edition, the Impact of Event Scale-Revised and the Short Form Health Survey to measure the quality of life of players who had retired due to concussion. After assessing a total of 76 players, they found that retired concussed ice hockey players had a low quality of life and high post-traumatic stress, particularly the players with a high concussion symptom burden.

## Maximizing discovery across the globe

*Concussion* utilizes a number of services to help support its researchers and maximize the visibility and impact of work. These include Publons (rewarding peer review), Altmetrics (tracking online discussion of articles, such as via social media or news outlets) and Dimensions (tracking citations). Download, Dimensions and Altmetrics statistics are shown on article pages, meaning authors can track the success of their articles.

To increase the reach of our articles we are keen to utilize the power of social media and post about every new publication on our Twitter page. If you do not already, be sure to follow our Twitter account (@fsgconcussion) for the latest *Concussion* articles, as well as relevant news and updates from the field at large. Alternatively, why not sign up for the journal eTable of Contents alert to ensure that you never miss the publication of an article. Information for how to sign up can be found on the homepage of our website.

## Our editorial board & contributors

We are hugely thankful to our Editorial Board for their help in growing *Concussion*, be that in an ambassadorial, advisory or authorship role. Our board comprises experts from across the globe with a wide range of expertise across the field of concussion research. If you would be interested in joining our Editorial Board, please get in touch; we would be delighted to hear your input.

We would also like to thank all the authors and peer reviewers for their contributions in 2020, without whom Volume 5 would not have been possible – we look forward to working with them again in 2021.

## Update from The Drake Foundation

It will come as no surprise that 2020 has thrown some challenges our way at The Drake Foundation and for the wider field of sports and concussion research. From the adapting of research protocols to account for a year of lockdowns and social distancing, to the cancelation of our annual symposium, it is certainly not been business as usual this year. Nonetheless, our work and that of our collaborators has found a way to continue and adapt to a COVID-19 world – updates on this year’s activities below.

### Adapting to a COVID-19 world

With organized sport postponed and universities largely closed during the UK’s first lockdown, so too affected were several of our research studies. For example, the complete pause to the end of the 2019/2020 season Premier League football (soccer) fixtures meant that our study with the University of Birmingham (UK) investigating biomarkers of concussion had to pause. This study [8] is led by Prof Tony Belli and Dr Patrick O’Halloran and includes the collection of saliva and urine samples from Premier League football players who sustain a concussion during a match, as well as nonconcussed controls. These samples are then tested using the Birmingham Concussion Test to measure miRNAs as biomarkers to indicate whether the brain has suffered injury. We hope that in the future, this test has the potential to assist in return-to-play decisions and could be used across sports, from grassroots to professional, in addition to military and other frontline settings. Given the pause to football matches in spring this year, sample collection was delayed through 2020, though we hope to pick up pace again throughout the 2020/2021 season.

However, while some studies were forced to pause completely throughout lockdown, others found ways to adapt: for example, the HEADING (Health and Ageing Data IN the Game of football) study [9]. Launched in 2019, the HEADING study is recruiting 300 former elite football players in order to examine the link between a history of concussion and heading the ball with neurodegenerative disease and long-term cognitive function. This study has seen some of the most major shifts due to the pandemic, with the research team having to think of new ways to recruit and then assess participants. While in-person assessments were the standard in the original study protocol, the research team rapidly pilot-tested a new virtual assessment protocol and found it delivered comparable results to in-person assessments, meaning the majority of participant assessments could continue throughout the year in a COVID-secure manner.

Despite the delays and adjustments throughout 2020, our research teams continue to inspire us with their proactive and dedicated approach to furthering our understanding of sport-related health and disease. We would like to take this opportunity to sincerely thank all our study researchers and collaborators for their diligent efforts in such a challenging year.

### Media storm

The latter months of 2020 saw a surge in media and public attention on the link between head impacts in sport and neurodegeneration, with football and rugby gaining most momentum in the UK press.

It was the sad news of the diagnoses of dementia in many of the England 1966 World Cup-winning squad that sparked a renewed and (at the time of writing) sustained focus on the relationship between a career in professional football and later cognitive decline. Following a surge in press coverage on football and dementia, a similar story soon followed in rugby. At the time of writing, several stories from former players – mostly in their 40’s – had just come to light, with the players sharing their heartbreaking recent diagnoses of early-onset dementia and likely chronic traumatic encephalopathy. In line with these stark and deeply personal stories, calls have also been made for sporting bodies to review practices to protect current and future players, as well as support those already suffering from dementia and other neurodegenerative diseases.

In 2019, the FIELD study [10] demonstrated that footballers have a greater risk of developing many neurodegenerative diseases than the general population. Although, the mechanisms behind this relationship require further research, as well as further research into the incidence of neurodegenerative disease in rugby players, there is no denying the strong indication that a history of sport-related head impacts in both football and rugby leads to an increased risk of neurodegenerative disease.

The Drake Foundation is itself funding several studies in both football and rugby, and supports the wider awareness and discussion of this critical topic. We encourage engagement from sporting bodies in this discussion and hope that we see evidence-led changes to sport and player support in order to inform and protect both current and future generations of players.

### The year ahead

Following a disrupted 2020, we look forward to what looks set to be a busy 2021. We anticipate the publication of data from a number of our studies, as well as a continued deepening of the conversation around sport and concussion. We also very much hope that we will be able to host our annual UK Sports Concussion Research Symposium in person again.

You can find out more about our ongoing studies and symposium by visiting our website [1].

## Conclusion

Back in March, as with the rest of the world, the Future Science Group team transitioned to a working-from-home model and we are proud to have been able to maintain our usual high quality of work outside of our standard office-based setting, with no slowdown in terms of article production or issue compilation. In fact, the past year has been one of the best so far for the journal, with an increase in submissions, publications and readership from 2019. We only hope that this trend continues as we enter 2021 and the journal continues to go from strength to strength.

We would like to finish by once again thanking all of the authors and reviewers who helped to make Volume 5 possible. Our readers remain central to the success of our journal and we welcome any feedback. Please do not hesitate to contact us with any suggestions for what you would like to see featured or any article proposals of your own. We welcome a wide range of unsolicited article proposals, so please do get in touch for more information.
